# The roles of COX-2 in protozoan infection

**DOI:** 10.3389/fimmu.2023.955616

**Published:** 2023-02-16

**Authors:** Xinlei Wang, Jie Chen, Jingtong Zheng

**Affiliations:** ^1^ Department of Clinical Laboratory, The Second Hospital of Jilin University, Jilin University, Changchun, China; ^2^ Institute of Theoretical Chemistry, Jilin University, Changchun, China; ^3^ Department of Pathogenobiology, College of Basic Medical Sciences, Jilin University, Changchun, China

**Keywords:** COX-2, protozoan infection, protozoa, AA, PGs

## Abstract

Protozoan diseases cause great harm in animal husbandry and require human-provided medical treatment. Protozoan infection can induce changes in cyclooxygenase-2 (COX-2) expression. The role played by COX-2 in the response to protozoan infection is complex. COX-2 induces and regulates inflammation by promoting the synthesis of different prostaglandins (PGs), which exhibit a variety of biological activities and participate in pathophysiological processes in the body in a variety of ways. This review explains the roles played by COX-2 in protozoan infection and analyzes the effects of COX-2-related drugs in protozoan diseases.

## 1 Introduction

Protozoan infection is one of the most common parasitic infections in humans and plays a very important role in worldwide morbidity and mortality (1). Protozoan infections are found around the world but are mainly concentrated in developing countries. The most important diseases in humans include malaria (2), leishmaniasis (3), Chagas disease (4), and giardiasis (5). Malaria is a life-threatening disease caused by *Plasmodium*, with parasites transmitted to humans through the bite of infected female *Anopheles* mosquitoes (3). World Health Organization (WHO) statistics show that five parasites are the main causes of malaria in humans, with *Plasmodium falciparum* (*P. falciparum*) and *Plasmodium vivax* (*P. vivax*) posing the greatest malaria threats (https://www.who.int/). Combined, these species caused 241 million malaria cases in 2020, with 627,000 malarial deaths (6). Leishmaniasis is a neglected tropical disease and the second leading cause of parasite-associated death, with 700,000 to 1 million new cases diagnosed each year (7). There are three main forms of leishmaniasis: visceral leishmaniasis (VL; also known as black fever and the most serious leishmaniasis), cutaneous leishmaniasis (the most common form), and mucocutaneous leishmaniasis. Left untreated, VL results in death in more than 95% of cases (8). Chagas disease is one of the most important diseases. It is a neglected tropical disease that has a high public health impact in the area. People can become infected through vector-borne transmission, congenital transmission, blood transfusions, organ transplantation and other transmission routes. Even in developed countries such as Germany, less than 1% of affected people receive adequate treatment (9). *Giardia lamblia* (*G. lamblia*) is the most common intestinal parasite worldwide (10). Immune-compromised individuals and undernourished children in developing countries are the most prone to severe manifestations of untreated giardiasis (11). The prevalence of *Giardia* in developing countries is estimated to range from 20% to 30% due to unsafe water supplies, ineffective environmental sanitation, and poor personal hygiene (12).

Despite several initiatives to reduce the incidence of protozoan disease, including treatment of giardiasis with the antibiotic metronidazole (13); treatment of malaria with artemisinin (14); and improvements in water, sanitation and hygiene conditions, protozoan infection remains a serious global health concern. Furthermore, drug resistance is a growing problem in veterinary medicine and may develop in humans; for example, metronidazole resistance has been reported in *Endamoeba histolytica* (*E. histolytica*) (15), *Trichomonas vaginalis* (*T. vaginalis*) (16) and *G. lamblia* (17). Clearly, there is a pressing need to develop new methods to control protozoan infection. One possible way to meet this need is to strengthen our understanding of the interactions between protozoa and hosts. Moreover, here, in addition to the four most relevant diseases, we also discuss other diseases, such as toxoplasmosis and babesiosis.

## 2 The role played by cyclooxygenase-2 in different diseases

After the protozoa invade the host organism, the protozoa and the host immune system are destined to fight. The result of this not only determines the fate of the parasite itself but also determines whether the host can survive and recover. The result of the fight depends on the location of the parasite within the host. Some intracellular parasites (for example, *Plasmodium*, *Leishmania*, *Trypanosome* and *Toxoplasma*) can enter and even reproduce in the host cells, but the host can destroy the infected cells. Extracellular parasites (e. g., *Giardia*), which are parasitic in the intestinal lumen, can be excreted due to the failure to adhere to epithelial cells or the destruction of cells after adhesion. Prostaglandin (PG) products play an important role for pathogens, and they are involved in many processes, such as inflammation, platelet aggregation, etc.

**Figure 1:**
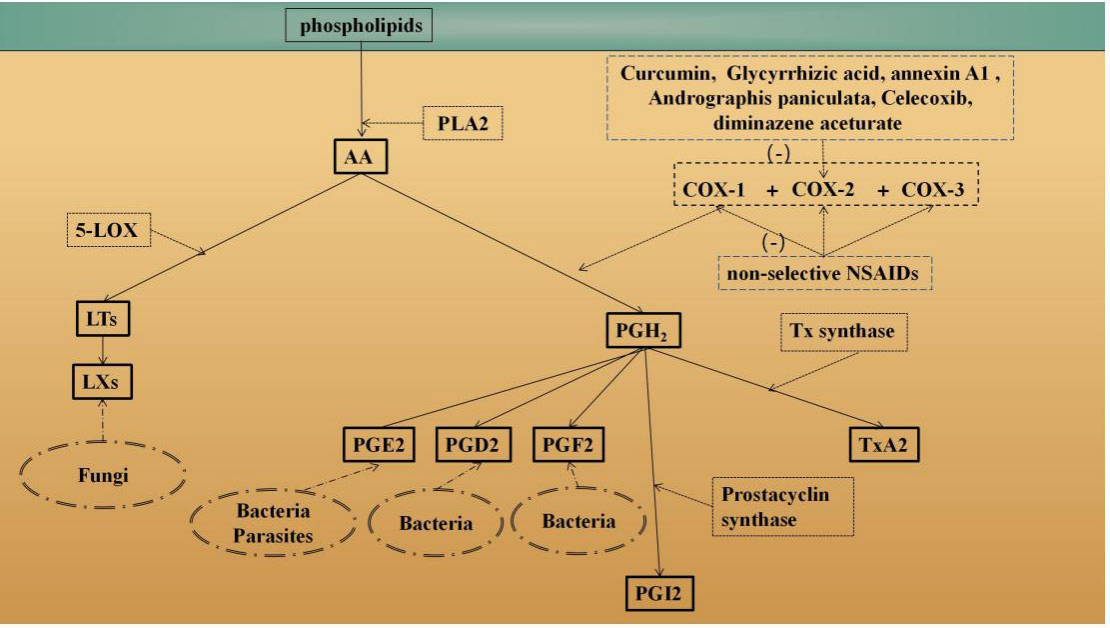
Overview of the AA metabolism pathways. Phospholipase enzymes (such as PLA2) can release AA from membrane-bound phospholipids (19) COX is the rate-limiting enzyme in prostaglandin-endoperoxide synthesis. AA can be metabolized by COX to PGH2, which is metabolized into four different PGs and tromboxane (Tx) (20–23). For example, prostacyclin synthase catalyzes the isomerization of PGH2 to form PG12 (20). TxA2 is formed via PGH2 by Tx synthase. In addition, AA can also produce inflammatory substances, such as leukotrienes (ET), through the 5-lipoxygenase (5-LOX) pathway (23). Different parts of the AA degradation pathway that occur in different pathogens (such as fungi, bacteria parasities) are also shown (24–27). Moreover, how anti-protozoal drugs are though to operate controlling COX-2 are also shown (28–34).

COX is the rate-limiting enzyme in prostaglandin-endoperoxide synthesis (18). It can metabolize arachidonic acid (AA) to form various PG products (**Figure 1**) (19–34). PGs play an important role in the regulation of human physiology and are involved in many processes, such as inflammation (35), platelet aggregation (36) and tumor development (37). COX is expressed in the following three isoforms: epoxygenase-1 (COX-1), COX-2 and COX-3 (38). The COX-1 enzyme was first identified in 1976 (39). The mouse gene (Ptgs1) encoding COX-1 was first isolated in 1993 (40). COX-1 is mainly expressed in blood vessels, interstitial cells, smooth muscle cells, and platelets (24), and after stimulation by growth factors or hormones, its expression level is increased 2-4-fold. Studies have shown that overexpression of COX-1 is associated with the development of various carcinomas (41, 42). COX-2 was identified in 1991 (43), and its discovery led to an understanding of the differences in PGs in normal function and disease. The gene (Ptgs2) encoding COX-2 is located on chromosome 1 (44). COX-2 is predominantly expressed in parenchymal cells in many tissues except the heart (24). Upon stimulation with IL-1, TNF, lipopolysaccharide (LPS), cAMP or other inflammatory factors, the COX-2 expression level can increase by approximately 80-fold, promoting a high rate of PG-endoperoxide and triggering an inflammatory response. PGs produced by COX-2 have multiple biological activities and can participate in pathophysiological processes in the body through various pathways (45, 46). The expression of COX-3 in human body is tissue specific, and COX-3 is common in the cerebral cortex and the heart. The typical remission period of chronic inflammatory diseases such as rheumatoid arthritis may be related to the expression of COX-3 (25).AA can be metabolized by COX into PGH2, which is then metabolized into four different PGs and tromboxanes (Txs), such as TxA2. For example, prostacyclin synthase catalyzes the isomerization of PGH2 to form PGI2. TxA2 is formed from PGH2 by Tx synthase. In addition, AA can also produce inflammatory substances through the 5-lipoxygenase (5-LOX) pathway, such as leukotrienes (LTs).

In tumor-related studies, PGE2 has been shown to be a proinflammatory cytokine and is overexpressed in a variety of human malignancies (47, 48). In various pathways, PGE2 binds to prostaglandin receptor (EP) in cancer cells, inducing tumorigenesis or promoting tumor progression (49). In intestinal tumors, overexpression of COX-2 contributed to the production of aberrant levels of PGE2 in epithelial cells. PGE2 plays various roles in cancer progression, including proliferation, migration, and immune escape. Tumor cells can acquire migratory capacity, which promotes metastatic colonization (50). Notably, ω-6 polyunsaturated fatty acids (PUFAs) were found to enhance the metastatic potential of gastric cancer cells *via* COX-2/PGE2 (51, 52). Thus, PGE2 has been shown to be a conventional target with pleiotropic effects in cancer onset and progression and can be used to develop novel potential treatments with clinical implications.

In addition, COX-2 plays an important role in vascular remodeling (53). COX-2 is an induced enzyme in the synthesis of PG intermediates, and its expression is closely related to vascular remodeling. Different induction factors upregulate the expression level of COX-2 by activating different signaling pathways, which subsequently activate different PGs, such as PGE2 or PGI2, and their respective receptors EP or IP to regulate vascular remodeling with the same or opposite effects. Moreover, regulator of calcineurin 1 (Rcan1) may be an endogenous negative regulator of COX-2 expression and activity, maintaining normal contractility and vascular stiffness in the aorta and mesenteric arterioles by inhibiting calcineurin and NF-κB pathway activation, respectively (54).

Alternatively, COX-2 plays an important role in endometrial-related lesions (55). Studies have shown that treatment with PGE2 and the PTGER2 agonist butaprost can induce the proliferation of bovine endometrial epithelial cells (bEECs). However, bEEC proliferation was attenuated by the PTGER2 antagonist AH6809 and CDK inhibitors (56).

Therefore, COX-2 plays an important role in many pathological processes, such as inflammation, coagulation, cell growth and tumor development. Because COX-2-related reports in protozoan infections are rare, in this review, we present an analysis of the function of COX-2 in different protozoan infections as reported in recent years and evaluate the correlations and differences.

## 3 Intracellular parasites

### 3.1 COX-2 in Chagas disease

#### 3.1.1 The azards of Chagas disease

*Trypanosoma cruzi* (*T. cruzi*), the main pathogen that causes Chagas disease, is estimated to infect approximately 6 to 7 million people worldwide (57). In Latin America, transmission to humans is mainly established through triatomine vectors (58). However, oral transmission, blood transfusion, maternal-neonatal transmission and organ transplantation are also known transmission routes (59). Hotez et al. (60) found that the prevalence of Chagas disease increased by 16% from 2000 to 2017. The WHO established World Chagas Disease Day in 2020 (April 14th) to call attention to Chagas disease (https://www.who.int/).

#### 3.1.2 Role played by COX-2 in Chagas disease

Chronic Chagas disease cardiomyopathy (CCC) is one of the most common types of chronic myocarditis in the world, with symptoms ranging from mild to severe cardiac remodeling with associated inflammation, fibrosis, arrhythmias and thromboembolisms, which may cause congestive heart failure and sudden death (61). Because of its important role in regulating inflammation and fibrosis in the heart, COX-2 has attracted great interest in the context of Chagas disease (62).

Studies have shown that macrophages and other innate immune cells form the first line of defense to inhibit parasite reproduction after parasite infection, as indicated by the immediate increase observed in the expression of proinflammatory cytokines and the production of the highly cytotoxic oxidant peroxynitrite (ONOO-) (63). Inhibition of COX activity may increase NO levels and thus restore antiparasitic activity in macrophages. Recently, obtained evidence suggests that COX is involved in the *T. cruzi* invasion process during infection (64). In the early stages of *T. cruzi* interaction with host cells, the parasites regulate cellular metabolism to enhance their own survival (65). Furthermore, although the effects of ROS on parasites are complex, some nonimmune cells, such as cardiomyocytes, respond to *T. cruzi* infection by producing ROS. In fact, several studies have identified mitochondria as the primary sources of ROS in *T. cruzi*-infected cardiomyocytes (66, 67). Typically, disruption of the mitochondrial membrane potential or loss of mitochondrial membrane structural integrity adversely affects the electron transport chain and causes increased mitochondrial ROS (mtROS) production (66). ROS production can then increase the nuclear localization of NF-κB and increase the NF-κB-mediated transcription of inflammation-related genes to promote an inflammatory response (68). Notably, when cardiomyocytes were injured, NF-κB activation induced the upregulation of COX-2 expression, and inhibition of COX-2 activity reduced the parasite-induced transcription of proinflammatory factors and enhanced ROS activity, conferring protection on cardiomyocytes (69).

#### 3.1.3 Drugs used to treat Chagas disease regulate COX-2 expression

Multiple drugs are currently available for the treatment of Chagas disease, including aspirin, benznidazole, and nifurtimox (4, 70). Among these drugs, aspirin triggers resolvin D1 production during the early chronic phase of *T. cruzi* infection, which modulates systemic infection levels and inflammatory responses in cardiac tissue and reduces immune cell infiltration, cardiomyocyte hypertrophy, fibrosis and the parasite load in heart tissue (70). Moreover, curcumin treatment in mice with acute Chagas disease improved mouse survival and prevented the activation of associated inflammatory processes in the heart (28). The curcumin mechanism of action may include inhibition of the Ca^2^+/NFAT-dependent pathological COX-2/mPGES-1/PGE2 pathway in trypanosome-infected cardiomyocytes, potentially conferring cardioprotection in infected mice (62). Moreover, inhibition of PGE2 synthesis with aspirin synergistically enhanced the activity of nitrofurantoin and benznidazole in infected RAW 264.7 cells (70, 71).

In conclusion, the role played by COX-2 in Chagas disease may be related to the attenuation of increased NO levels, negatively affecting the antiparasitic activity of macrophages. It may also be related to a reduction in the ROS content in cardiomyocytes. From the perspective of protecting cardiomyocytes, the use of anti-inflammatory drugs can exert a protective effect on cardiomyocytes by regulating inflammation and fibrosis in the heart.

### 3.2 COX-2 in leishmaniasis

#### 3.2.1 The hazards of leishmaniasis

Since the WHO first recognized leishmaniasis as a neglected tropical disease and the second leading cause of parasite-related death, more than 1 billion people in 98 countries still face a risk of contracting this disease (https://www.who.int/). Depending on the type of leishmaniasis, symptoms may include fever, weight loss, and partial or total destruction of the mucous membranes of the nose, mouth, and throat (72).

In 2020, most VL cases were diagnosed in Brazil, Ethiopia, India, Kenya, Somalia, and Sudan (https://www.who.int/). According to one estimate, 500,000 patients are first infected with VL each year, and approximately 12 million people are infected with *Leishmania* parasites (73). Dogs are considered the main urban sources of *L. infantum*, which strongly parasitizes canine skin, and people in contact with infected dogs can be infected.

#### 3.2.2 Role played by COX-2 in leishmaniasis

*Leishmania* can upregulate COX-2 expression and PGE2 synthesis (20), and PGE2 can regulate the microbicidal properties of macrophages in a manner mediated by NO_2_ (74). A COX-2 inhibitor (NS-398) has been reported to reduce the parasite load in peritoneal macrophages of mice infected with *Leishmania donovani* (*L. donovani*) (75). Moreover, antibodies against serine protease (SP) inhibited COX-2-mediated PGE2 synthesis and reduced ROS production while enhancing the expression of T helper 1 cell (Th1) cytokines, such as IL-12 (76). Since defense against *Leishmania* relies on Th1 inflammatory responses (77, 78), SP is suggested to be an important novel target for treatment of VL.

*De novo* linoleic acid synthesis in *Leishmania* is required for parasite survival in the extracellular promastigote and the intracellular amastigote stages (79). Due to the anti-inflammatory properties of linoleic acid, it may regulate COX-2. Western blotting showed that infection with a linoleic acid-deficient *Leishmania* (KO) mutant led to increased phosphorylation of NF-κB p65, IkB and IKKb in RAW264.7 cells. Similarly, RAW264.7 cells infected with KO *Leishmania* showed a significant increase in COX-2 expression and TNF-α secretion compared with other cell lines. Therefore, we speculate that COX-2 and TNF-α expression in macrophages may be increased by the activation of the NF-κB signaling pathway. In addition, after incubation for 2 h to 6 h, the adhesion rate of promastigotes was the same in all groups. However, the internalization rate of promastigotes in the KO group at 24 h was lower than that in the other groups. Most infected macrophages in the control group still contained parasites after 72 h of incubation; however, after 72 h of incubation, the number of parasites in the KO group was significantly smaller than that in the control group. When RAW264.7 cells were pretreated with BSA-bound linoleic acid, the KO group was found to have a higher infectious capacity than the control group. Thus, KO parasites are more likely to be killed within macrophages (79).

#### 3.3.3 Drugs used to treat leishmaniasis regulate COX-2 expression

Resveratrol is found in various medicinal plants (33). In addition to its cytotoxic, antifungal, antimicrobial and cardioprotective effects, resveratrol also inhibits COX-1 and COX-2 activity (80). Recent *in vitro* and *in vivo* assays demonstrated that resveratrol at all concentrations and resveratrol nanoemulsions exert important inhibitory effects against *Leishmania* (81). This effective antiparasitic activity may be related to potential mitochondrial membrane depolarization, increased plasma membrane permeability, and interference with cell cycle progression (82).

Glycyrrhizic acid (GA) is the main active ingredient of licorice and has played an important role in traditional Chinese medicine and research since ancient times (29). Recently, GA has been found to have antiparasitic activity (83). Experimental GA treatment *in vitro* resulted in enhanced expression of inducible NO synthase (iNOS) and DUSP4 and inhibition of COX-2 expression. GA treatment of infected macrophages enhanced the expression of IL-12 and TNF-α, which was accompanied by the downregulation of IL-10 and TGF-β expression (83, 84). GA increased the macrophage effector response by inhibiting COX-2-mediated PGE2 synthesis in *L. donovani-*infected macrophages (85). GA also reduced the parasite load in the liver and spleen and increased T-cell proliferation in BALB/c mice infected with *Leishmania* (79, 86).

In conclusion, although COX-2-related signaling pathways involved in *Leishmania* infection have not been extensively studied, genetic mutations have indicated that COX-2 expression may be increased by NF-κB signaling pathway activation. Therefore, drugs that can inhibit NF-κB signaling expression, COX-2 expression and PGE2 synthesis might be a future direction in drug research.

### 3.3 COX-2 in toxoplasmosis

#### 3.3.1 The hazards of toxoplasmosis

Toxoplasmosis is a common disease caused by *Toxoplasma gondii* (*T. gondii*), a parasite with a high prevalence in the tropics (87). The *T gondii* life cycle largely progresses in cats (88). Most *T. gondii* infections lead to mild symptoms, but immunocompromised patients tend to have a poor prognosis. Therefore, toxoplasmosis can be a significant disease in patients infected with human immunodeficiency virus and with acquired immunodeficiency syndrome (HIV/AIDS) or other immunosuppressive diseases (89).

#### 3.3.2 Role of COX-2 in toxoplasmosis

Many studies have demonstrated a role of COX-2 and PGE2 in *T. gondii* infection (32, 90–92). Notably, COX-2 and PGE2 promote the survival of different pathogens in host cells, induce the replication and dissemination of pathogens, and downregulate the immune response (93, 94). COX-2 expression has been shown to be significantly increased in *T. gondii* infection, confirming that this parasite is a potent inducer of COX-2 (95). *T. gondii*-infected macrophages may exhibit modulated AA content (a COX-2 substrate) mediated through the calcium signal transduction pathway, and the PKC-dependent COX-2 metabolic pathway has been shown to regulate PGE2 synthesis (91). Moreover, the induction of lipid droplet (LD) formation by *T. gondii* in host cells was found to be closely associated with COX-2 expression. LD formation primarily maintains *T. gondii* survival but is also important for the production of inflammatory mediators (96). An LD core consists of phospholipids and neutral lipids, and when cells detect parasites, these LDs release AA, which can be converted to PGE2 *via* COX-2 activity (97).

Moreover, since *T. gondii* tachyzoites pass through the placenta and can reach fetal tissue during pregnancy, which can cause serious birth defects that exert impacts into adulthood, the study of *T. gondii* replication in trophoblast cells and chorionic cells is important (98). Studies have shown that the COX-2 expression levels and PGE2 production levels are significantly increased, proinflammatory cytokine (IL-6 and MIF) expression is induced, and the level of anti-inflammatory cytokines (IL-4 and IL-10) and the number of LDs are increased in toxoplasmosis (32). Thus, COX-2 facilitates intracellular *T. gondii* proliferation in human trophoblast cells and human chorionic villi, downregulates proinflammatory mediator expression and increases LD production in cells, thereby inhibiting immune responses in the environment where the parasite lives.

#### 3.3.3 Drugs or proteins used to treat toxoplasmosis regulate COX-2 expression

Many anti-*Toxoplasma* drugs have been developed, and the combination of sulfadiazine and pyrimethamine is currently the gold standard for the treatment of toxoplasmosis (99). In addition, spiramycin and some Chinese medicine ingredients, such as curcumin and artemether, can significantly inhibit the proliferation of *Toxoplasma* (100–102). Among these medicines, the effect of curcumin on *T. gondii* may be similar to its effect on *T. cruzi*. Recent studies have shown that annexin A1 (ANXA1) expression in cytotrophoblasts is decreased after *T. gondii* infection. ANXA1 is a calcium-dependent phospholipid-binding protein that mediates glucocorticoid action, inhibits PG synthesis, and limits the abundance of COX-2. Treatment with ANXA1 reduces the parasitism rate of placental explants in the third trimester of pregnancy (32). *In vitro* cultured trophoblast cells provide a cellular model for studying the mechanism by which various substances cross the placental barrier; furthermore, these cells provide the cytological basis for studying the pathogenesis of mother-to-child transmitted diseases. Since *T. gondii* tachyzoites reach the fetal tissue through the placenta during pregnancy, it is important to investigate *T. gondii* replication in trophoblast and chorionic cells (27). It has been reported that COX-2 inhibitors reduced *T. gondii* replication in trophoblast and chorionic tumor cells and altered the expression of inflammation-associated cytokines (32). For example, in BeWo cells (human chorionic tumor cells), COX-2 inhibitors induced an increase in proinflammatory cytokines (IL-6 and MIF) and reduced the expression of anti-inflammatory cytokines (IL-4 and IL-10). In HTR-8/SVneo (human chorionic trophoblast) cells, COX-2 inhibitors (meloxicam or celecoxib) induced an increase in IL-6 and nitrite and a decrease in IL-4 and TGF-1 levels (32). In addition, COX-2 inhibitors reduced the number of LDs in the two cell types.

In conclusion, the increase in COX-2 expression and PGE2 production induced by *T. gondii* infection in macrophages and other cells is clear. However, the mechanism directing this increase is not clear, with multiple pathways possibly regulating the increase in COX-2 expression and PGE2 production. Studies targeting LDs may be a direction for future research.

### 3.4 COX-2 in malaria

#### 3.4.1 The hazards of malaria

A 2021 study reported a total of 241 million malaria cases worldwide in 2020, and the total number of malaria-related deaths worldwide was 627,000. Of these, 95% of malaria cases and 96% of malaria deaths occurred in Africa.

*Plasmodium* mainly infects two types of hosts, mosquitoes and humans; it undergoes sexual reproduction in mosquitoes and asexual proliferation in humans (103). *Plasmodium*-carrying *Anopheles* mosquitoes transmit the parasite by biting humans (104). Then, the parasite migrates through the dermis and enters the blood, where it is carried to the liver (105). The parasite then enters hepatocytes and develops schizonts. The schizonts are released from hepatocytes into the bloodstream, where they invade red blood cells (106). They begin the next phase of their life cycle in these red blood cells, progressing through ring stages to the trophozoite site and then the schizont stage (107). Malaria clinical outcomes range from the complete absence of symptoms to severe disease and death. Children under the age of 5 years and pregnant women are the most vulnerable groups affected by malaria (108).

#### 3.4.2 Role of COX-2 in malaria

Pregnant women are particularly susceptible to malaria due to immunological changes during pregnancy (109). Placental malaria caused by *Plasmodium berghei* (*P. berghei*) and *P. falciparum* can upregulate PG synthesis by increasing COX-2 enzyme activity (110). COX-2 and PGs cause uterine contractions and can therefore induce miscarriage or preterm birth (31). Thus, regulating COX-2 and PG levels is very important to fetal health.

Moreover, COX-2 regulates severe malarial anemia (SMA). SNP variations and their genetic combination in the COX-2 promoter have been reported to be related to the longitudinal risk of malaria, SMA and all-cause mortality in children living in areas with high *Plasmodium falciparum* transmission (111). Notably, in contrast to the high expression of COX-2 in pregnant mice infected with *P. berghei*, PGE2 levels in the monocytes of mice which infected with *P. falciparum* were significantly associated with the increase in plasma IL-10 levels (112). In *P. falciparum* infection, the induction of high PGE2 levels by COX-2 is an important host defense mechanism (113). *In vitro* studies have shown that reduced PGE2 production is caused by the downregulation of COX-2 expression due to the associated production of parasite-produced hemozoin (114). Hence, a central feature of SMA pathogenesis was found to be the systemic inhibition of PGE2 production, and reduced systemic PGE2 levels during infection are at least partially mediated by leukocyte phagocytosis of hemozoin (115). *In vitro* and *in vivo* experimental results suggest that inhibition of COX-2-mediated PGE2 production is associated with TNF-α overproduction in children with malaria (115).

Furthermore, in *P. falciparum*-transmitted areas, children with malaria often present with an increased incidence of other infections, such as bacterial infections and HIV-1 (116, 117). Therefore, the COX-2 expression and PGE2 production in children with malaria and children coinfected with bacteria or HIV-1 were also analyzed. The results showed that the COX-2 expression levels in peripheral blood and bicyclo-PGE2/creatinine levels in plasma were significantly reduced in coinfected children compared to levels in malaria monoinfected children. Furthermore, inhibition of circulating bicyclo-PGE2 was significantly associated with reduced hemoglobin levels in children with either malaria mono- or coinfection (112), suggesting that bicyclo-PGE may be a marker and mediator of malaria pathogenesis.

COX-2 also plays a very important role in cerebral malaria (CM). COX-2-derived PGE2 is negatively correlated with the disease severity of CM caused by *P. falciparum* (118). Altered blood brain barrier integrity and cytokine expression patterns are key determinants of central nervous system lesion formation in patients with CM caused by *P. falciparum* (119). Moreover, in clinical trials, the peripheral blood of CM patients showed altered PG concentrations (112). PG synthesis is controlled by many types of cyclooxygenases, and COX expression has been found to play a key role in immune regulation, hemostasis, and inflammatory reactions in multiple pathologically altered brain tissues. COX expression in CM patient brains was determined through immunohistochemistry. The accumulation of COX-2-expressing endothelial cells and astrocytes was detected in CM brain samples (120).

Similar to elevated COX-2 expression in infected pregnant mice, COX-2 expression was shown to be elevated in C57BL/6 mouse models of CM established with *P. berghei* (121). In a comparison study with CM-susceptible CBA mice, C57BL/6 mice and malaria-resistant BALB/c mice, all three mouse types responded to *Plasmodium*. The mice exhibited increases in the number of LDs in macrophages and a significant inflammatory response. Furthermore, the expression of COX-2 and 5-lipoxygenase (5-LOX) was enhanced in the brain tissue cells and blood vessels of C57BL/6 mice and CBA mice. However, neither COX-2 nor 5-LOX was expressed in the brain tissue cells or blood vessels of BALB/c mice. In the macrophages of infected BALB/c mice, PPAR expression levels were increased in the nucleus and decreased in the cytoplasm, suggesting PPAR-γ nuclear translocation (122, 123). PPAR-γ translocation to the nucleus may result in downregulation of proinflammatory cytokine production (124), delaying disease severity in BALB/c mice; this possibility may explain the increased infection resistance in BALB/c mice.

#### 3.4.3 Drugs used to treat malaria regulate COX-2 expression

*P. berghei* caused elevated COX-2 expression in pregnant mice, but *Andrographis paniculata* (AS201-01) tablets significantly reduced placental COX-2 expression and PG production in pregnant mice infected with *P. berghei* (31). The anti-inflammatory activity of AS201-01 may inhibit iNOS and COX-2 expression by inhibiting p38/MAPK signaling pathway activation (125). AS201-01 can increase TGF-β and decrease TLR-4 expression and the apoptotic index, ultimately inhibiting *P. berghei* growth (31).

Studies have shown that although aspirin and celecoxib are effective in *T. cruzi*-induced diseases, caution must be taken when they are used to treat malaria. The reason for this may be related to platelets, because human platelets are important for a variety of *Plasmodium* parasites. Thus, the inhibition of platelet function by platelet inhibitors such as aspirin may also eliminate the positive effects (126). Erythrocytic parasite analysis indicates that platelet-associated parasite killing is characterized by intracellular erythrocytic accumulation of platelet factor 4 (PF4) (127). This function of PF4 is critically dependent on the Duffy antigen (Fy) receptor binding to PF4. Previous studies have shown that evolutionary selection of African Fy -negative alleles provides protection against *P. vivax* infection (128). Meanwhile, recent studies showed that Fy binds the platelet effector molecule PF4 and is required for platelet-mediated killing of *P. falciparum* (129). Platelet killing of *P. falciparum* requires platelet-iRBC contact, the release of PF4, and the binding of PF4 to Fy receptors. The mechanism may be that the release of PF4 upon direct platelet-iRBC contact allows access to *P. falciparum via* the Fy (129).

In recent years, artesunate has been used as a first-line treatment for severe malaria in adults and children. The use of artesunate has been shown to reduce mortality in patients with severe malaria (130). Artemisinin binds to a very wide range of parasitic proteins and can affect diverse organelles and cellular processes, including hemoglobin endocytosis, glycolysis, protein synthesis and degradation, and cell cycle regulation (131). Artemisinin-induced decreases in COX-2 expression have mainly been observed in cancer (132). Artesunate significantly inhibited gastric cancer cell proliferation in a time- and dose-dependent manner and induced the apoptosis of gastric cancer cells in a dose-dependent manner, and this apoptotic effect was found to be associated with decreased COX-2 expression (133). Furthermore, artesunate prevents neuroinflammation in BV2 microglia by interfering with the NF-κB and p38/MAPK signaling pathways (134). However, whether COX-2 regulates neuroinflammation in malaria has not been extensively investigated.

In conclusion, hemozoin is an insoluble crystalline pigment produced by *Plasmodium* after digestion of host hemoglobin within erythrocytes. After rupture of infected erythrocytes, hemozoin is released into the bloodstream and is phagocytosed by circulating monocytes and tissue macrophages (135). Experiments with cultured blood monocytes have shown that hemozoin increases the release of cytokines, such as the proinflammatory cytokine TNF-α and the anti-inflammatory cytokine IL-10. Then, TNF-α induces high levels of persistent COX-2 gene expression (136), whereas reduced peripheral PGE2 biosynthesis induced by *Plasmodium* occurs through hemozoin-induced suppression of blood mononuclear cell COX-2 gene expression *via* increased IL-10 expression (113); hence, the regulatory effects of hemozoin on COX-2 expression may be complex.

### 3.5 COX-2 in babesiosis

#### 3.5.1 The hazards of babesiosis

Babesiosis, also known as redwater, is a blood protozoonotic disease transmitted by hard ticks (137). The clinical symptoms of acute bovine babesiosis are fever, limb weakness, anemia, and sometimes animal death (138). Babesiosis is endemic in many countries in Europe, Asia and Africa (139). Babesiosis is mainly transmitted between domestic animals and wild animals, but certain strains are zoonotic (140).

#### 3.5.2 Role of COX-2 in babesiosis

LDs are key regulators of not only inflammation and toxoplasmosis but also babesiosis. Increases in the number of liposomes during bovine Babesia infection have been reported to be associated with Babesia strains and the expression of COX-2 and other enzymes (141, 142). The attenuated strain R1A (LA) and toxic strain S2P (LV) induced TNF-α and IL-6 secretion by mouse peritoneal macrophages after infection with both parasites. Moreover, the LA strain elevates liposome content and COX-2 expression, which is associated with TLR2 and TLR6 expression, respectively (141, 142).

#### 3.5.3 Drugs used to treat babesiosis regulate COX-2 expression

Different *Babesia* species differ in their drug susceptibility. Large canine *Babesia* species, such as *Babesia canis* and *Babesia rossi*, are sensitive to imidocarb dipropionate and diminazene aceturate, but small species, such as *Babesia gibsoni* and *Babesia conradae*, are relatively resistant to these drugs. Therefore, combination treatment with hydroxynaphthoquinone atovaquone and the antibiotic azithromycin is usually used (143). Azithromycin and other antibiotics with antiprotozoal properties mainly target the apicoplast, a residual plastid found in protozoa, and eventually cause parasite death (144). Moreover, tafenoquine may be a highly useful drug to treat *B. microti* infection (145). The regulatory effect of these drugs on COX-2 has been less frequently reported than the effects of other drugs.

Studies have shown that diminazene aceturate exerts a regulatory effect on COX-2. For example, the local use of diminazene aceturate in the treatment of endotoxic uveitis downregulated the mRNA expression levels of inflammatory factors and mediators, such as COX-2 and iNOS, in the iris and ciliary body. Azithromycin inhibited PGE2 synthesis in human leukocytes by inhibiting the expression of cPLA2, COX-1, and COX-2 mRNA, suggesting an anti-inflammatory mechanism of action (146).

In conclusion, the drug sensitivity of different *Babesia* species may be related to the apicoplast or the proteins encoded by the mitochondrial genome. For example, certain apicoplasts or proteins encoded by the mitochondrial genome can be targeted by antibiotics such as ciprofloxacin and rifampicin. However, only anti-*Babesia* drugs, such as diminazene aceturate, have shown a regulatory effect on COX-2; however, whether other drugs have a regulatory effect on COX-2 expression remains to be determined.

## 4 Extracellular parasites

### 4.1 COX-2 in giardiasis

#### 4.1.1 Hazards for giardiasis

Giardiasis is caused by the protozoan parasite *Giardia* (147); these parasites are transmitted through the fecal-oral route, usually after ingestion of contaminated water or food or contact between individuals (148). Approximately half of *Giardia*-infected children are asymptomatic. Other children develop either acute or chronic diarrhea (149). Metronidazole and tinidazole are the preferred drugs for treating giardiasis (13), but resistance to common anti-*Giardia* drugs has increased in recent years (150). Therefore, the search for new molecular targets against *Giardia* has become urgent.

#### 4.1.2 The role of COX-2 in giardiasis

COX-2 plays a very important role in inflammation during *Giardia* infection (149). Studies have shown that the levels of COX-2 and proinflammatory cytokines (TNF-α, IL-6 and IL-1) are increased in the intestinal tissue of infected mice. Moreover, in a coculture with *Giardia*, the expression levels of COX-2 and proinflammatory cytokines were significantly increased in J774A.1 macrophages (151). These results indicate that *Giardia* can induce the upregulation of COX-2 expression levels in both macrophage infection and mouse infection models, suggesting a possible role played by COX-2 in *Giardia* infection. Furthermore, pretreatment with the COX-2 inhibitor NS398 attenuated the upregulated expression of these proinflammatory cytokines, suggesting that *Giardia*-induced upregulation of inflammatory cytokine expression may be mediated through COX-2. Administration of SB203580 (an inhibitor of p38), SCH772984 (an inhibitor of ERK1/2) or JSH-23 (an inhibitor of NF-κB) after infection with *Giardia* led to COX-2-mediated inflammatory factor expression activation *via* the NF-κB and p38/ERK1/2/MAPK signaling pathways (151).

The effect of COX-2 on the apoptosis of intestinal epithelial cells (IECs) may be another key pathogenic factor in giardiasis. After treatment for *Giardia* infection, the amount of nitric oxide (NO) released from IECs gradually decreased. Moreover, a COX-2 inhibitor diminished cell viability, exacerbated the reduction in NO release, and increased the cell apoptosis rate (152). In contrast, COX-2 overexpression induced by the agonist rebamipide inhibited the apoptosis of IECs induced by *Giardia* infection. Cell apoptosis is closely related to the MAPK/AKT/NF-κB signaling pathway. For example, the MAPK/AKT/NF-κB signaling pathway has been reported to modulate COX-2 expression (149). First, the p38 inhibitor SB202190, ERK1/2 inhibitor SCH772984, and AKT inhibitor MK-22062 were found to block the upregulated COX-2 expression induced by *Giardia* infection, revealing an association between p38/ERK/AKT signaling and COX-2. Then, the TLR4 inhibitor TAK-242 was found to significantly block p38 phosphorylation and COX-2 expression in IECs infected with *Giardia*, suggesting that TLR4-dependent p38 signaling plays a role in regulating COX-2 expression. Moreover, in the p38-NF-κB signaling pathway, COX-2 expression was inhibited by the NF-κB p65 inhibitor JSH-23.

#### 4.1.3 Drugs used to treat giardiasis regulate COX-2 expression

The antiprotozoan activity of indazole derivatives has been recently reported (153). The indazole core is a very important basic structure in pharmacochemistry, and it is widely used to regulate the inflammatory response (154, 155). Considering that protozoan infections are associated with the inflammatory response, researchers designed a group of 2H indazole derivatives to treat parasite infections. Among these derivatives, compound No. 18 showed 12.8-fold greater activity than metronidazole against *Giardia*. Interestingly, compound No. 18 also showed inhibitory activity against COX-2 *in vitro* (153). The design of novel antiparasitic compounds with additional COX-2 inhibitory properties may be an interesting direction for future drug production. However, other studies have shown that *Giardia*-triggered apoptosis may increase IEC permeability and aid in the development of giardiasis through the action of the protease Giardipain-1 (156); thus, antiapoptotic therapy is thought to be an effective strategy to mitigate giardiasis without harming uninfected tissues.

Overall, inhibition of COX-2 activity can promote a reduction in the IEC activity induced by *Giardia*. Moreover, increasing the production of reactive oxygen species (ROS) and reducing the release of NO aggravates the apoptosis of IEC cells. Therefore, inhibition of COX-2 activity does not exert a protective effect on IECs but plays a role in promoting apoptosis. In contrast, COX-2 overexpression can reduce IEC apoptosis induced by *Giardia*. Increased ROS levels and decreased NO levels have been identified as stimuli leading to IEC apoptosis (157, 158). The levels of ROS/NO may be influenced by COX-2 expression, which is regulated by MAPK/AKT/NF-κB signaling (159, 160).

However, it is worth noting that IECs are renewed every 3-5 days (161, 162); therefore, *Giardia* trophozoites must always adhere to new IECs to avoid being eliminated by intestinal movement (163). In this case, apoptosis of some IEC cells may play a positive role in giardiasis by preventing *Giardia* from colonizing the intestinal epithelia.

In conclusion, we found that the effects of COX-2 on giardiasis may be complex. Although inhibition of COX-2 activity does not protect IECs, it promotes apoptosis in IECs. However, the results of promoting IEC apoptosis may be diverse. On the one hand, IEC apoptosis can prevent parasite infection by preventing *Giardia* colonization. On the other hand, it may also cause epithelial damage and aggravate inflammatory reactions. Therefore, the current research seems to be focused on exploring which method (COX-2 overexpression or COX-2 inhibition) is more favorable for controlling giardiasis. However, the results of current studies are not consistent.

### 4.2 COX-2 in Acanthamoeba Keratitis (Acanthamoebiasis)

#### 4.2.1 The hazards of Acanthamoeba Keratitis

*Acanthamoeba* spp. are pathogenic and opportunistic free-living parasites that cause AK and granulomatous amoebic encephalitis (GAE) in immunocompromised individuals (164). The biological and pathogenic features underlying these opportunistic protozoa are not fully understood. Therefore, a focus on the relationship between the parasites and hosts is needed. The symptoms of AK are mostly redness, photophobia, tears, conjunctival congestion and eye pain, and AK is usually misdiagnosed as herpetic, bacterial or fungal keratitis (165).

#### 4.2.2 Role of COX-2 in AK

In the eyes, PGE2 formation may be attributed to both COX-1 and COX-2 expression. PGE2 is one of the most extensively studied PGs. PGE2 is critical for producing fever and pain and in neurotransmitter modulation. The role played by PGE2 in the eyes has been studied, especially in the context of low intraocular pressure (IOP) (166, 167). Increased PG production may exacerbate inflammation and downregulate the immune response and cytokine production (IL-1, IL-2, IFN-γ and TNF-α), key determinants controlling disease severity and outcome. Moreover, the synthesis of PGs, especially PGE2, is increased under parasite stimulation (168). PGE2 signals binding to the G protein-coupled receptor EP4 can stimulate adenylate cyclase (AC) and then promote cAMP production. Elevated cAMP expression can stimulate cyst formation (169). Thus, increased generation of PGs affects cyst formation (169).

#### 4.2.3 Drugs used to treat AK regulate COX-2 expression

Agahan et al. (170) reported three cases of AK that were successfully treated with nonsteroidal anti-inflammatory drug (NSAID) eye drops. NSAIDs inhibit COX activity. Studies have shown that diclofenac sodium and indomethacin significantly inhibit *Acanthamoeba castellanii* (*A. castellanii*) growth (171). Moreover, both diclofenac sodium and indomethacin inhibited cyst formation. In 2015, Aqeel et al. (172) observed that the inhibitory effect of G protein-coupled receptors affected the growth of *A. castellanii in vitro*. These findings, together with our observations of the inhibitory effect of NSAIDs on *A. castellanii* growth, suggest that inhibition of COX expression reduces PG synthesis and thus downregulates G protein-coupled receptor activity, leading to downstream cascade blockade and ultimately causing cell cycle arrest. Acetaminophen is a weak inhibitor of PG synthesis, which may explain its ineffectiveness against *A. castellanii*. NSAIDs are frequently used in clinical practice, and they may be informative for the design and improvement of therapeutics and may lead to better prevention strategies when combined with other antiamoebic drugs.

## 5 Conclusion

In this review, we discussed the effects of COX-2 expression in host cells in several different parasitic diseases, including Chagas disease, leishmaniasis, giardiasis, trichomoniasis, amebiasis, malaria and babesiosis (**Table 1**). For intracellular parasitic parasites, the role of COX-2 may be related to the regulation of inflammatory factor expression in the immunological cells of infected animals. For example, aspirin can trigger resolvin D1 production in the early chronic stage of *T. cruzi* infection, and resolvin D1 regulates systemic infection levels and inflammatory responses in heart tissue by reducing immune cell infiltration, cardiomyocyte hypertrophy, fibrosis, and parasite load in heart tissue. Notably, the effects of different *Plasmodium* species on COX-2 may be complex. For example, *P. berghei* can induce increased COX-2 expression, but *P. falciparum* can reduce COX-2 expression. Extracellular parasites such as *Giardia* can cause increased COX-2 expression in macrophages. However, the inhibition of COX-2 activity can change its adhesion ability; for example, inhibiting the COX-2 pathway can change IEC cell activity, promote the apoptosis of IEC, and affect the subsequent pathogenic ability of the parasite. It is important to note that changes in COX-2 activity may also affect the cell cycle. Therefore, in addition to its role in cancer, COX-2 may also inhibit different stages of cell cycle progression, such as that of Acanthamoeba. Many inflammatory factor-related signaling pathways are also involved in these diseases, such as the NF-κB and p38/ERK1/2/MAPK pathways. Therefore, clarifying the effect of COX-2 on parasites may lead to the development of better antiparasitic drugs. It should be noted that the most widely available COX-2 inhibitors, NSAIDs, have limited application in the treatment of certain diseases, such as cancer, due to certain side effects. Therefore, further studies are needed to identify drugs that can effectively regulate COX-2 or PGE2 and are better for use against parasites.

**Table 1:** COX-2 in different parasitic diseases.

Disease	*Parasite*	Tissue or cell of action	Drugs that regulate COX-2 expression	Reference
Giardiasis	*Giardia*	Intestinal tissue	2H-idiazole derivatives	(153)
Chagas Disease	*Trypanosoma*	Macrophages and cardiomyocytes	Curcumin Resolvin D1	(62, 74)
Leishmaniasis	*Leishmania*	Macrophages	Resveratrol Glycyrrhizic acid	(34, 83)
Toxoplasmosis	*Toxoplasma*	Macrophages	Curcumin ANXA1	(32, 100)
Malaria	*Plasmodium*	Monocytes	AS201-01 Artesunate	(31, 130)

## Author contributions

JZ performed the majority of the research and data analyses and helped draft the manuscript. XW and JC analyzed and interpreted the raw data. All authors contributed to the article and approved the submitted version.
